# Antenatal care in practice: an exploratory study in antenatal care clinics in the Kilombero Valley, south-eastern Tanzania

**DOI:** 10.1186/1471-2393-11-36

**Published:** 2011-05-20

**Authors:** Karin Gross, Joanna Armstrong Schellenberg, Flora Kessy, Constanze Pfeiffer, Brigit Obrist

**Affiliations:** 1 Swiss Tropical and Public Health Institute, Basel, Switzerland; 2 University of Basel, Basel, Switzerland; 3 London School of Hygiene and Tropical Medicine, London, UK; 4 Ifakara Health Institute, Dar es Salaam, United Republic of Tanzania; 5 University of Basel, Institute of Anthropology, Basel, Switzerland

## Abstract

**Background:**

The potential of antenatal care for reducing maternal morbidity and improving newborn survival and health is widely acknowledged. Yet there are worrying gaps in knowledge of the quality of antenatal care provided in Tanzania. In particular, determinants of health workers' performance have not yet been fully understood. This paper uses ethnographic methods to document health workers' antenatal care practices with reference to the national Focused Antenatal Care guidelines and identifies factors influencing health workers' performance. Potential implications for improving antenatal care provision in Tanzania are discussed.

**Methods:**

Combining different qualitative techniques, we studied health workers' antenatal care practices in four public antenatal care clinics in the Kilombero Valley, south-eastern Tanzania. A total of 36 antenatal care consultations were observed and compared with the Focused Antenatal Care guidelines. Participant observation, informal discussions and in-depth interviews with the staff helped to identify and explain health workers' practices and contextual factors influencing antenatal care provision.

**Results:**

The delivery of antenatal care services to pregnant women at the selected antenatal care clinics varied widely. Some services that are recommended by the Focused Antenatal Care guidelines were given to all women while other services were not delivered at all. Factors influencing health workers' practices were poor implementation of the Focused Antenatal Care guidelines, lack of trained staff and absenteeism, supply shortages and use of working tools that are not consistent with the Focused Antenatal Care guidelines. Health workers react to difficult working conditions by developing informal practices as coping strategies or "street-level bureaucracy".

**Conclusions:**

Efforts to improve antenatal care should address shortages of trained staff through expanding training opportunities, including health worker cadres with little pre-service training. Attention should be paid to the identification of informal practices resulting from individual coping strategies and "street-level bureaucracy" in order to tackle problems before they become part of the organizational culture.

## Background

There is little evidence that antenatal care (ANC) prevents maternal mortality [1-3]. However, the potential of antenatal care for reducing maternal morbidity and improving newborn survival and health has been widely acknowledged [4]. The antenatal period provides excellent opportunities to reach pregnant women with prophylactic medication, vaccinations, diagnosis and treatment of infectious diseases, as well as with health education programs [5]. Proven effective antenatal interventions include serologic screening for syphilis, provision of malaria prevention, anti-tetanus immunization and prevention of mother-to-child-transmission of HIV [6,7]. Provision of advice during antenatal care about potential pregnancy complications and danger signs, and information on how to seek medical care, are viewed as key strategies to reduce delay in seeking skilled care [1,8]. Moreover, a positive association between the level of care obtained during ANC and skilled delivery care has been reported [9]. Emphasizing the quality instead of the quantity of visits, the Focused Antenatal Care (FANC) model promoted by the WHO reflects this new understanding of the role of ANC [10].

In Tanzania, the Ministry of Health and Social Welfare implemented the FANC policy in 2002 and used it for cascading health worker training on a central, regional and district level [11,12]. The FANC model emphasizes goal-oriented and women-centred care by skilled providers [11]. Activities of the new model include the early detection of danger signs and referral; therapeutic interventions known to be beneficial; and alerting pregnant women to emergencies and instructing them on appropriate responses [10]. In fact, one of the main goals of the new model is to strengthen the information component through individual health education and counselling [10,13].

However, quality assessments of antenatal care services provided to pregnant women raised questions about health workers' performance: practice often diverges from the standards required in the guidelines [14-18]. In Tanzania, national data from 2004/05 indicated that less than half of all women received information on signs of pregnancy complications, had urine samples taken or were given a full dose of preventive anti-malaria chemotherapy [19]. Other recent studies examining single antenatal care programs or routine ANC provision in Tanzania reported in particular the poor quality of technical aspects such as clinical and laboratory examinations [14,16,17,20] or drug administration [14,17]. Boller et al. [14], who assessed quality of care in public and private ANC clinics in Dar es Salaam, found that guidelines were frequently not respected and diagnostic examinations were not carried out by health workers. At 12 minutes for first visits and 6.5 minutes for return visits, consultation times were short and differed significantly from the required time anticipated according to the FANC guidelines (42 minutes and 32 minutes respectively) [18]. Health problems may thus often be missed [21]. Moreover, there are reports of poor counselling and inadequate health education of pregnant women [16-18,22,23] or negative health worker attitudes [24].

Although inadequate health workers' performance has been widely described, determinants of poor performance are not fully understood [25]. In many studies national guidelines serve as a "gold standard" to assess observed health workers' performance during patient consultations [26]. However, recent qualitative studies emphasize the importance of comprehending the complex context in which guidelines are put into practice. Mathole et al. [27] and Walker and Gilson [28], for example, assessed the implementation of policy changes in Zimbabwe and South Africa and illustrated health workers' difficulties in handling the changes due to resource shortages and poor policy implementation. They showed that health workers developed informal practices in order to cope with the high demand for their services and the difficult working situation. Two studies from Tanzania and the UK illustrated how peer pressure, perceived patients' preferences and team support lead clinicians to take decisions based on constructed "mindlines" that are the result of day-to-day practice rather than evidence-based knowledge [29,30].

The aim of this exploratory study is to investigate the interplay between policy, context and practice and its influence on antenatal care provision in four rural ANC clinics in south-eastern Tanzania. First, it examines how health workers' ANC practices relate to the national FANC guidelines. Second, reasons for health workers' practices are explored from health workers' points of view. Finally, the study's insights and their potential implications for antenatal care provision in Tanzania are discussed.

## Methods

### Study area

Data for this study were collected in health facilities during research visits of one week per facility in July 2008 and during short one-day follow-up visits in April 2009 in the Kilombero and Ulanga Districts, Morogoro Region in south-eastern Tanzania. The study area comprised the 25 villages of the 'Health and Demographic Surveillance System' that has been described extensively by other authors [31-34]. The Tanzanian public health system consists of a dense network of dispensaries, health centres and hospitals. At the time of the study, two public health centres and ten dispensaries (7 public and 3 private not-for-profit) provided Reproductive-and-Child-Health (RCH) care services in the research area on a weekly or daily basis from Monday to Friday. Two district hospitals served as referral hospitals. The local health system runs a cost-sharing scheme from which pregnant women and children under five years of age are exempted.

Four public health facilities were selected in the study area: both of the health centres (HC) and one selected dispensary (D) from each district. The selection of the dispensaries was based on the criteria of 1) daily RCH service provision and 2) high numbers of pregnant women attending the RCH clinic based on patient registers.

### Data collection

**Figure 1:**
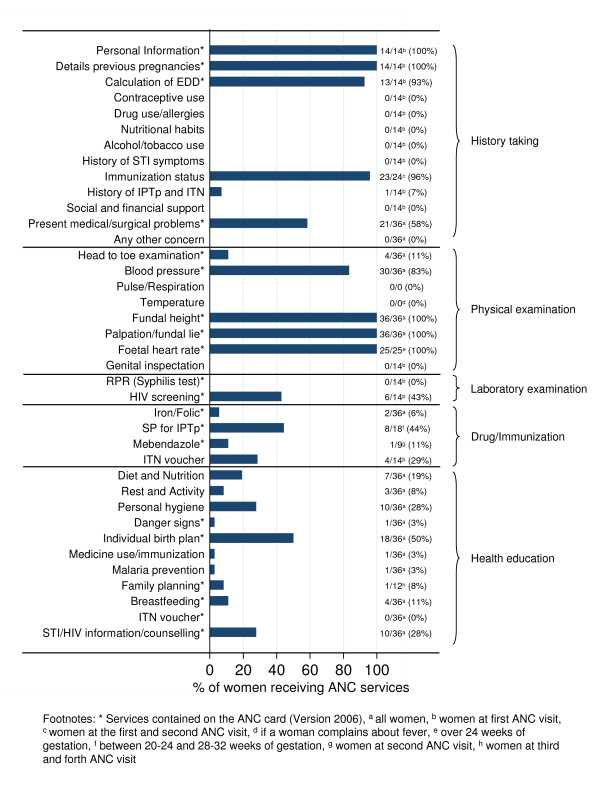
**Proportion of pregnant women receiving each of the 38 services recommended by the guidelines**.

The present study used qualitative methodology including 4 elements: 1) participant observation of daily RCH clinic procedures, 2) structured observation of ANC consultations, 3) informal conversations with pregnant women and health workers and 4) in-depth interviews with the five health workers available at the RCH clinics at the time of the study. Data collection was carried out in Swahili at each health facility over a one-week period by one of the investigators (KG). She was supported by a research assistant who could help with nuances of the language. In the four health facilities, 39 ANC consultations were selected for observation by convenience sampling. ANC consultations were spread over the whole week and included consultations of women attending for the first time as well as return visits. The number of observed consultations per health worker ranged from 3 to 21, depending on the number of women attending per facility. Three women were excluded from the sample since they did not receive any services, and thus their consultations could not be observed. Two of them attended on the "wrong" day and one woman came with an early pregnancy that could not be confirmed. The three women were sent home and told to come again another day. This led to a final sample of 36 observed ANC consultations. Structured observation was used to record services delivered during the ANC consultations. A checklist including 41 recommended services was developed on the basis of the Tanzanian FANC guidelines [11]. Three services delivered at the laboratory facilities were later excluded because they could not be directly observed. This led to a final list of 38 recommended services on which data were collected (see Figure 1).

Because of the health workers' high work load, the participant observers became involved in administrative work and registering clients. Informal conversations with the health workers during and after work helped to understand clinic procedures and to clarify questions that had arisen during the observations. Notes were taken during the observations and conversations and were elaborated the same day in descriptive field notes [35] in collaboration with the research assistant.

Towards the end of the week, in-depth interviews were conducted with the five health workers who had been present at the time of the study. The interview guidelines explored contextual factors influencing health workers' ANC practices such as health workers' training and position, their perceived work problems, work expectations and interaction with their patients, colleagues and supervisors. All in-depth interviews were tape-recorded with health workers' permission.

### Data analysis

The in-depth interviews were transcribed and translated into English by two research assistants fluent in English and Swahili. One of us (KG) reviewed the transcripts and original recordings and discussed ambiguities with the research assistants.

For data analysis, data from the structured observation of 36 ANC consultations were compared with the FANC guidelines [11] and the ANC card. For each of the 38 services it was determined whether according to the FANC guidelines the women should have received the specific service considering her gestational age and/or number of ANC visits. This was then compared with the structured observations of ANC consultations.

Data from the in-depth interviews, the participant observations and informal conversations were used to contextualize and validate the findings from the structured observations. Data analysis was guided by a mix of inductive and deductive category building and was completed using MAXqda2 (VERBI Software, Marburg, Germany). In the in-depth interviews, the most prevalent themes raised by the health workers were coded into categories using qualitative content analysis [36] and tested in the further analysis of the interviews. The same categories were applied to the field notes of the observations and informal conversations in order to check their validity. Additionally, analysis of all data sources was guided by the researchers' interest in how rules and regulations determine health workers' practices. In order to explore differences in service delivery between and within health facilities, information on the identified themes was cross-tabulated for comparison between and within the health facilities. Questions arising during data analysis were addressed in follow-up and feedback visits at the four health facilities in April 2009.

### Ethical considerations

In conformity with the Helsinki Declaration, this study was discussed and approved by the district coordinators for Reproductive and Child Health (RCH) and staff in -charge were asked for permission to conduct the study at their facilities. Oral or written consent was obtained from all pregnant women and health workers participating in the study after explaining the purpose of the study to them and informing them of their right to withdraw at any time.

The study received clearance from the Tanzanian National Institution for Medical Research as part of the ACCESS Programme (NIMR/HQ/R.8c/Vol. I/66). The study was also approved by the two review boards of the Swiss Tropical and Public Health Institute (STPH), formerly known as Swiss Tropical Institute (STI), and the Ifakara Health Institute (IHI), formerly known as Ifakara Health Research and Development Centre (IHRDC).

## Results

### ANC in practice

The ANC clinics officially opened at 8am and closed at 3.30pm. Health workers encouraged pregnant women to arrive early in the morning, but service delivery usually did not start on time either due to the late arrival of the pregnant women or due to the health workers being busy with other activities, such as attending children. While ANC return visits tended to take a few minutes and only consisted of abdominal examinations, blood pressure measurements, and the administration of Sulphadoxine-Pyrimethamine (SP) and other drugs, pregnant women's first ANC visits were time-intensive. They were organized along the five thematic components of service provision stipulated by the FANC guidelines: 1) history taking, 2) physical examination, 3) laboratory examinations, 4) drug administration and immunization and 5) health education. Figure 1 provides an overview of the services delivered to pregnant women in comparison with the requirements of the national FANC guidelines and Table 1 gives a descriptive account of a typical morning at one of the ANC clinics.

**Table 1:** Description of a typical morning at one ANC clinic based on field notes

Activity	Routine ANC procedures
	We arrived at the health facility at 9:30 am. At the RCH clinic, around forty mothers and their children were waiting outside the examination room for the auxiliary nurse to vaccinate the children. The auxiliary nurse was attending the children alone because the nurse midwife had left the facility a month ago. She weighed and vaccinated them, filled in the cards and gave health education to the mothers. Three pregnant women who had arrived in the morning were waiting outside. It was their first visit to the ANC clinic. At 2 pm the auxiliary nurse started to attend them.

**History taking**	In the attendance room the auxiliary nurse started to collect personal information from the pregnant women and to ask them about their history of previous pregnancies and illnesses. She registered the information on the ANC cards and the health facility register. Using the date of the last menstruation she calculated the expected delivery date. The other two pregnant women listened quietly.

**Physical examination**	Although the auxiliary nurse initially wanted to postpone the height and length measurement to the women's next visit, she changed her mind and went to measure and weigh the women. Then, she invited the women to the examination room for the physical examination. One at a time, each woman went to the separate delivery room and lay down on the bed. The auxiliary nurse measured fundal height, listened to foetal heart sounds and palpated the child's position.

**Drug administration/immunization**	Then, the women received Tetanus vaccines and got their blood pressure measured. Finally, the women were asked to get water from the drug dispensing room to swallow SP.

**Laboratory investigations**	The women were told to come back on the 24^th ^of the same month to test for Syphilis because the test would then be conducted for all pregnant women. None of the women were tested for HIV/AIDS. The auxiliary nurse explained that she was not able to perform the test because the only person who was trained had gone for training. She told them to get tested in another health facility.

**Health education**	Then, the health education started. The nurse was first sitting on a chair but got up saying that she was used to standing while giving the health education. She disseminated the health messages in a didactic manner: standing in front of the women, telling them what they should do and asking questions to check the women's attention. Often the women did not respond to her questions. She emphasized the importance of starting ANC attendance early. Then she started to talk about hygiene and stressed that women should keep themselves and their clothes clean. She reminded the women to put small savings to the side in order to be prepared for the delivery and for potential emergencies requiring transport to the hospital. She explained what supplies they would need for the delivery and emphasized the importance of giving birth at the health facility and not with a traditional birth attendant (TBA). She stressed that TBAs lack supplies and experience. She explained the Tetanus schedule to the women with the help of the Tetanus card and asked them to come back to the health facility for the postpartum care.

History taking was usually conducted individually, although in one health facility women were asked these personal questions in the presence of the other women. Women's information was recorded in the health facility register and on women's ANC cards. During the physical examination, the main activity observed was the abdominal examinations, including manual palpation of the foetus, measurement of the fundal height, the fundal lie and listening to the foetal heart rate that was performed for all women. Most of the women had their blood pressure measured; however, genital examination and check of body temperature, pulse and respiration were not conducted at any of the selected health facilities. A few cases of oedema were recognized during the examination and addressed. Laboratory tests such as for urine, haemoglobin and the blood group were conducted in the three health facilities where special laboratory infrastructure was available. HIV and Syphilis were tested at the ANC clinics using rapid tests. However, at some places the tests were only conducted on a weekly or monthly basis in order to decrease work load. After examination, women were given SP for Intermittent Preventive Treatment in pregnancy (IPTp), Mebendazole and iron/folate tablets. However, administration was often constrained by stock-outs as illustrated by Table 2. Health education sessions were usually held either at the beginning or at the end of the ANC visit, but they were only conducted for women attending the ANC clinic for the first time. The main topics were STI and HIV prevention, personal hygiene, diet and nutrition. Information on how to plan and prepare for delivery were hardly addressed here but were brought up during the physical examination.

**Table 2:** Availability of laboratory tests and drugs at the time of study

	D1	**D2 **^**a**^	HC1	HC2
**Laboratory examinations**				

**Urine test**	✔	✘	✔	✘ ^b^

**Haemoglobin**	✔	✘	✔	✘ ^b^

**RPR (Syphilis test)**	- ^d^	✔	✘ ^b^	✘ ^b^

**Blood group/Rhesus factor**	✔	✘	✔	✔

**HIV screening**	✘ ^b^	✘ ^c^	✔	✘ ^b^

**Malaria tests**	✔	✔	✔	✔

**Drug/Immunization**				

**Iron/Folic**	✘ ^b^	✔	✔	✘ ^b^

**SP**	✘ ^b^	✔	✔	✔

**Tetanus**	✔	✔	✔	✔

**Mebendazole**	✘ ^b^	✘ ^b^	✔	✔

**Hati Punguzo vouchers**	✘ ^b^	✘ ^b^	✔	✘ ^b^

^a ^laboratory infrastructure not available

^b ^lack of drugs or supplies (f.e. gloves, reagents)

^c ^lack of training to perform the test

^d ^no data available

All in all, Figure 1 demonstrates that service delivery varied widely and was generally not according to FANC guidelines: 12 of the services recommended by the FANC guidelines were not given to any women, a further 18 services were given to 3%-58% of women and eight services were given to over 80% of women.

### Understanding health worker practices

This section explores reasons for health workers' non-compliance with the FANC guidelines by looking at the context and health workers' practices. Four major themes emerged from the data analysis: 1) absenteeism and lack of training, 2) lack of resources, 3) ANC cards as "working guidelines", and 4) informal rules and routines.

#### Absenteeism and lack of training

At the time of the study, out of eight health workers routinely working in the four selected RCH clinics only five were present; three of them were trained to provide RCH services (two MCH Aides who worked jointly at one health facility and one nurse midwife) and only one had been trained on the FANC guidelines. This reflects a problem prevalent at all four selected health facilities: staff shortages, absenteeism and lack of training on the FANC guidelines. Table 3 summarizes the availability and qualification of the health workers working at the selected RCH clinics and indicates whether or not they had received training on the FANC guidelines.

**Table 3:** Characteristics of the health workers working at the selected RCH clinics

**Type of health facility**^**a**^	Qualification of health workers (years of training)	Years of work experience	Availability of health workers and reason for absence	Training on the FANC guidelines
**D1**	Auxiliary nurse (1)	28 yrs	available	No

	Nurse midwife (5)	- ^b^	unavailable due to death in the family	Yes

**D2**	Auxiliary nurse (1)	16 yrs	available	No

	Nurse midwife (5)	- ^b^	unavailable due to staff turnover and delay of replacement	Yes

**HC1**	Certified nurse midwife (4)	24 yrs	available	No

	Nursing officer with diploma (6)	- ^b^	unavailable due to sickness	Yes

**HC2**	MCH Aide (2)	26 yrs	available	Yes

	MCH Aide (2)	16 yrs	available	No

^a ^Health Centre (HC), Dispensary (D)

^b ^Missing information (-)

According to the national staffing level guidelines, dispensaries should be staffed with five staff members (2 clinical officers, 2 public health nurses and 1 nurse attendant) [37]. However, in each of the two dispensaries selected, only three trained health workers were present at the time of the study. Hence, RCH services were provided by nurse auxiliaries, because the nurse midwives were absent due to staff turnover and the death of a close relative. Nurse auxiliaries receive a minimal training of one year and are reported as being the most inadequately skilled in identifying women's pregnancy conditions and understanding the national FANC guidelines [38]. However, often they have experience through many years of working in a dispensary or health centre.

In one of the health centres, a nurse midwife was providing services solely because her colleague (a nursing officer with a degree) was sick. In the other health centre, RCH services were provided by two experienced Mother and Child Health (MCH) Aides, a cadre trained to provide mother and child services at dispensaries and health centres. One of them had been trained on the FANC guidelines. Not surprisingly, health workers complained in the in-depth interviews about the lack of sufficient personnel and described their working situation as stressful.

*"If you would decide to stay a whole day at home [after delivering a baby during the night], there would be nobody here to do the work. Therefore, the nurse goes there (out-patient department) and returns here (RCH department) until she gets exhausted. Vaccines, children, pregnant women, patients, there is always someone"*. (Auxiliary nurse, D1)

Absenteeism not only left the remaining staff with a higher work load but also with responsibilities that often exceeded the qualification expected for their cadre. In particular, auxiliary nurses had to deal with the dilemma of either treating cases for which they were not adequately qualified or not delivering the services at all. In one case, an auxiliary nurse detected that one of the women had a problem with her breasts. She called the doctor who diagnosed a skin problem and referred her to the hospital. After the doctor had left, the auxiliary nurse uttered uncertainty about what drug to prescribe as the doctor had not advised anyone. In another dispensary, the auxiliary nurse was not allowed to perform HIV tests because she had not participated in the training seminar. A nurse midwife had been sent to the seminar, but she had left the facility in the meantime. Pregnant women were therefore referred to the next health facility at 30 km distance in order to be tested for HIV (Table 1).

Training on the FANC guidelines had been conducted in 2007, however, reportedly due to financial constraints, only one health worker per health facility could be invited. Moreover, health workers with minimal pre-service training were excluded from the training due to the plans of the Ministry of Health and Social Welfare to phase out this cadre (Personal communication, District RCH coordinators).

#### Lack of resources

All interviewed health workers expressed frustration with the given work situation at their health facilities. In particular, complaints arose about lacking drugs and supplies needed for laboratory investigations. Table 2 summarizes the availability of laboratory tests and drugs at the time of the study and shows that even at the three health facilities where laboratory infrastructure existed, some of the tests could not be performed due to stock-outs of supplies such as gloves or reagents. Drug shortages prevented the delivery of SP or Mebendazole used for the prevention of malaria and soil-born diseases among pregnant women. At one dispensary where SP was out of stock, pregnant women were sent to the nearby drug shop in order to buy SP. At two other health facilities, health workers reported that they manage to restock needed items by obtaining them thanks to established relationships with the staff of a neighbouring health facility or a drug shop.

*"If we have shortages of SP, we usually go to request it [at the district] or we go to the neighbouring health facility. If we run out of [SP] we go to ask there"*. (Nurse midwife, HC1)

*"If the supplies are available in the nearby drug shop, we run over to borrow them to get them without troubling people. Later on when the facility gets them they go to pay because we have a close relationship with the shop here. Apart from things like razors and gloves we buy small things there, if we don't have them"*. (MCH Aide, HC2)

User fees had been introduced in the early 1990s in the study area either in the form of consultation fees or prepayment (f.e. Community Health Funds) with the aim of enhancing facilities' ability to improve their quality of care. However, buying drugs from providers other than the Tanzanian Medical Stores Department (MSD), which is the official drug supplier, is mostly not an option, because funds from consultation fees are not foreseen for the purchase of drugs but rather for minor repairs, the purchase of kerosene or the payment of watchmen. Community Health Funds that could be used for the purchase of drugs are often not accessible to health facilities due to high administrative burdens.

#### ANC card as "working guidelines"

Observations and informal conversations showed that the FANC guidelines did not play a large role in guiding the daily work of the health workers. In three of the four health facilities, health workers did not know whether the FANC guidelines were actually available at the health facility or not. ANC cards, however, provided an important working tool for them. Health workers used the card as continuous patient documentation and registered personal information, physical examinations, laboratory tests, and drug and health education delivery. Pregnant women were supposed to bring the card to each visit, and the ANC card structured the delivery of the ANC services. Unfortunately, the ANC cards (Version 2006) only cover a subset of the services recommended in the FANC guidelines (see services marked with * in Figure 1). This might explain why some of the recommended services were not delivered to the women (see Figure 1 and Table 4). History-taking, for example, was reduced to those four elements for which information is requested on the ANC card. Other information, such as on contraceptive use, IPTp use and Insecticide Treated Nets (ITN) utilization, as well as on social and financial support, was not collected, since health workers were not able to register the data anywhere. Table 4 illustrates that health services for which information was requested on the ANC card were delivered far better than services recommended by the FANC guidelines but not listed on the ANC card.

**Table 4:** Consistency between information requested on the ANC card and service delivery

ANC card	Service delivery		
		n/N	%

Information requested on ANC card [N = 20]	Services delivered to at least 50% of the women	9/20	45
	
	Services not delivered to any woman	2/20	10

Information not requested on ANC card [N = 18]	Services delivered to at least 50% of the women	1/18	6
	
	Services not delivered to any woman	10/18	55

#### Rules and routines

In all selected health facilities, daily clinic activities were guided by informal rules and routines that had been introduced in order to cope with the perceived high work load. Although officially the selected health facilities provided ANC services daily, in reality pregnant women came for their first ANC visit only on certain days of the week. Health workers explained that specific attendance schedules had been introduced several years ago at the health facility level in order to cope with the increased work load due to the introduction of HIV tests. At some health facilities, schedules for laboratory tests existed in order to reduce the work load. In one of the dispensaries, syphilis tests were only offered on a monthly basis, while in another health facility HIV tests were performed on Thursdays only. Pregnant women were supposed to return to the ANC clinic on the specified day to get tested. Organizational rules had been created by the individual health facility teams without consulting the district authorities. As the district authorities promote daily ANC services, these informal rules not only conflicted with the national FANC guidelines but also with district aims.

Observation showed that the individual health workers enforced informal rules and routines more or less strictly. Although they were usually interacting with their clients in a friendly and joking manner, sanctioning of women was observed, especially in one health facility. An auxiliary nurse refused to examine some of the pregnant women, sent them back home or told them off because they did not obey the health facilities' organizational rules. On one occasion, the nurse did not attend a woman who made her first ANC visit on a day not scheduled for first attendees and told her to come back on the correct day. On another occasion, she scolded a woman who had lost her ANC card when her house caught fire. The auxiliary nurse asked her to prove the incident with a confirmation letter from the village leader if she wanted to get another ANC card for free. These examples illustrate that health workers were in a position to interpret and enforce existing informal rules in an individual way. By sanctioning the women for their non-compliant behaviour they demonstrated power and exerted hierarchical control over their clients. Whether health workers made use of this opportunity or not depended on their individual work motivation, their confidence in working skills and on their relationship with the women.

## Discussion

The findings of this exploratory study in four rural ANC clinics in south-eastern Tanzania confirm evidence from previous studies on poor quality of ANC provision in Tanzania and other countries [14,15,17-20,22,24,39]. Observation of ANC consultations revealed that the provision of ANC services varied widely and was not in accordance with the FANC guidelines; some of the services that pregnant women were supposed to receive were not delivered to any of the women, while others were given to nearly all women (see Figure 1). Performance during return visits was particularly poor. Consistent with previous quality assessments of Boller et al. [14] in public and private ANC clinics in Dar es Salaam, Sarker et al. [16] in the Rufiji District, and Gilson [20] in the Kilombero Valley, this study revealed critical gaps in clinical and laboratory examinations and drug administration. Furthermore, our results are supported by a study of von Both et al. [18] who found major discrepancies between current ANC practice and the requirements of the FANC guidelines, especially in health education and counselling.

This study was based on a small sample of four rural ANC clinics, and its results might not be applicable to other countries or even to other settings in Tanzania. Nevertheless, using a combination of different qualitative techniques, the study's in-depth exploration of health workers' practices and working context extends the available evidence. It offers new interesting and relevant insights for understanding determinants of health workers' ANC provision in a rural, resource-constrained setting that should be investigated at a larger scale.

First, the findings clearly demonstrated that in all four health facilities, lack of trained staff and absenteeism was critical. Out of eight health workers routinely working in the four selected RCH clinics, only five health workers were present at the time of the study. Among these, three health workers had the skills to provide MCH services (two MCH Aides working jointly in one of the health facilities and one nurse midwife) and only one had been trained on the FANC guidelines. This reflects not only a critical shortage of skilled health work force, but also raises questions about the implementation of the FANC guidelines. Given that only one health worker in the four selected health facilities had been trained on the FANC guidelines, non-adherence to the guidelines is no surprise. The low availability of skilled staff at the health facilities might not be representative of other regions as at national level a high proportion of pregnant women are reported to receive ANC services from nurse midwives (70%) [19]. At the same time, several studies revealed that understaffing of qualified health staff is worst in rural dispensaries of the public sector [40-42]. This study provided evidence that unskilled staff, left without the support of their absent colleagues, not only had to deal with a high workload but also to handle cases for which they were not trained. This could lead to frustration and put pregnant women's health at risk [43]. Efforts should, thus, focus on training all health workers on the FANC guidelines. Moreover, based on the study's finding that health workers who are least skilled are often highly experienced and need to take over the responsibilities of their trained colleagues when they are absent, this group should not be excluded from training opportunities. Instead, considering the critical shortage of adequately skilled health staff, efforts need to be made to enable them to adequately deliver the basic services required at the dispensary level. Providing them with prospects for training and career development not only has the potential to improve their skills but might additionally result in a positive spill-over effect of increasing their motivation to work in a rural setting [44].

Second, the study pointed to the important role that ANC cards played in health workers' daily provision of ANC services compared to the FANC guidelines. This is probably because of health workers' lack of training on the guidelines. In fact, ANC cards served as institutionalized "working guidelines" and adherence to the cards' instructions was high. Figure 1 demonstrates that ANC service delivery followed the items listed on the ANC cards but did not cover the whole spectrum suggested by the FANC guidelines due to differences between the ANC cards and the FANC guidelines. This finding ties in with insights of Rowe et al. [45] and Walter et al. [46] on the Integrated Management of Childhood Illness (IMCI) strategy. They report how differences between the national reporting system and the guidelines had a similar impact on the quality of IMCI diagnoses: health providers diagnosed and treated sick children narrowly because they based their diagnoses on the requirements of the Health Management Information System (HMIS) instead of the more complex IMCI guidelines. Eliminating discrepancies between the FANC guidelines and the ANC cards would provide health workers not only with user-friendly "working guidelines" but might also constitute an easy and promising approach to improve the performance of even those health workers who have never been trained on the FANC guidelines.

Third, the findings clearly confirmed the impact of the lack of material resources and health system failures on the quality of ANC provision. Health workers were struggling on a daily basis with stock-outs of laboratory supplies and drugs due to weak health infrastructure and health system failures. Policies introduced to mitigate health system failures, such as user-fee schemes, proved not to be functional. The study, thus, complements other studies on the impact of non-availability of resources on health workers' performance [25]. While some point out that the lack of resources might cause serious dilemmas for health workers' decision-making [27,43,47], others stress its negative impact on health professionals' work motivation [43,44,48]. As Reis et al. [49] show, it might also lead to health workers' discriminatory behaviour towards clients, if, for example, they have to attend HIV patients but lack protective and other materials to treat and prevent the spread of HIV.

Finally, our study contributes to evidence showing that health workers may react to a complex and often stressful working environment created by lack of training, staff shortages and resource constraints by adopting coping strategies in the form of predatory behaviour and brain drain [50,51] or with "street-level bureaucracy" [28]. This term was coined by Michael Lipsky [52] who emphasized on the one hand the critical role of front-line health workers in delivering public services, and on the other hand their struggle to cope with contextual factors such as lack of adequate organizational and staff resources. Hence, front-line workers, including health workers, develop and implement informal practices in order to cope with the high demand for their services and the difficult working situation. Informal practices are difficult to identify as they are usually not consciously reflected. As a result, few studies have examined them with regard to health care and especially ANC. An exception is Mathole et al. [27] who explored women's and health workers' attitudes towards the implementation of a new ANC package in Zimbabwe and also showed health workers introducing informal organizational rules in order to cope with a high work load. While difficult working conditions certainly force health workers to create routines that allow for mass treatment such as health education sessions in groups, there is also reluctance to change these long-established routines, for example by introducing more time-consuming individual counselling. Informal rules and routines often contradict official regulations and might be misused by individual health workers in order to demonstrate power and exert hierarchical control. They should therefore receive more attention within research. However, in some cases informal practices may also lead to positive outcomes. Our study indicates that health workers revealed a surprising ability to mobilize lacking drugs and supplies from alternative sources by drawing on established relationships with neighbouring health facilities and shops. Supportive supervision could have the potential not only to support positive outcomes but also to prevent dangerous consequences of informal practices.

## Conclusions

This rigorous though exploratory analysis gives summary measures of quality of ANC and reveals important determinants of health providers' (non-)compliance with the national FANC guidelines. Moreover, it provides a basis for initial lessons about how to strengthen ANC provision in a rural resource-constrained setting.

The study illustrates that for ANC services to be effective and meet standards, both trained staff and material resources are required. However, conditions in Tanzania are often insufficient, particularly in rural areas where resources are even more constrained. Factors influencing the quality of ANC provision may lie outside the control of health workers, and force them to come up with their own informal strategies to cope with the situation.

Improvements of working conditions should focus on the remedy of supply shortages and the strengthening of human resources. This means provision of opportunities for training and career development for those who belong to the least-trained health worker cadres. The high compliance with the ANC card reported in this study provides promising evidence that health workers' performance can even be good under constrained conditions. Performance targets need to be well defined, institutionalized and achievable and take the often difficult working context of health workers into account. Furthermore, researchers and policy-makers should give more attention to the detection and identification of informal practices caused by "street-level bureaucracy" and individual coping strategies. Problems caused by informal practices need to be tackled before they become part of the organizational culture. Regular supervision and participatory solution-finding are key strategies. Routine ANC provision must build on the social resources available in the health system. Thus, positive outcomes of health workers' coping strategies as observed in this study need to be fostered by supporting the exchange between peers and health facilities.

## Competing interests

The authors declare that they have no competing interests.

## Authors' contributions

KG was involved in the design and implementation of the study, field work, data management, analysis and interpretation of the data, and writing of the manuscript. BO, FK and JS supported the design of the study. JS, FK, CP, BO contributed to the manuscript. All authors have read and approved the final manuscript.

## Pre-publication history

The pre-publication history for this paper can be accessed here:

http://www.biomedcentral.com/1471-2393/11/36/prepub
